# PD‐1 expression affects cytokine production by ILC2 and is influenced by peroxisome proliferator‐activated receptor‐γ

**DOI:** 10.1002/iid3.279

**Published:** 2019-11-19

**Authors:** Banu Batyrova, Fien Luwaert, Panagiota Maravelia, Yuria Miyabayashi, Neha Vashist, Julian M. Stark, Sara Y. Soori, Christopher A. Tibbitt, Peggy Riese, Jonathan M. Coquet, Benedict J. Chambers

**Affiliations:** ^1^ Department of Medicine, Centre for Infectious Medicine (CIM), Karolinska Institute Karolinska University Hospital Huddinge Stockholm Sweden; ^2^ Department of Vaccinology and Applied Microbiology Helmholtz Centre for Infection Research Braunschweig Germany; ^3^ Department of Microbiology, Tumor and Cell Biology (MTC) Karolinska Institute Stockholm Sweden

**Keywords:** cytokine, ILC2, peroxisome proliferator‐activated receptor‐γ

## Abstract

**Introduction:**

Innate lymphoid cells (ILCs) can provide early cytokine help against a variety of pathogens in the lungs and gastrointestinal tract. Type 2 ILC (ILC2) are comparable to T helper 2 cells found in the adaptive immune system, which secrete cytokines such as interleukin 5 (IL‐5) and IL‐13 and have been found to play roles in host defense against helminth infections and in allergic responses. Recent studies have identified that programmed cell death protein 1 (PD‐1) and peroxisome proliferator activated receptor‐γ (PPAR‐γ) are highly expressed by ILC2. We examined whether PD‐1 plays a role in ILC2 function and whether there was any connection between PD‐1 and PPAR‐γ
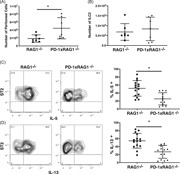

**Methods:**

To ensure that only innate immune cells were present, ILC2 cells were examined from RAG1^−/−^ and PD‐1^−/−^xRAG1^−/−^ mice under steady‐state or following inoculation with IL‐33. We also tested ILC2 generated from bone marrow of RAG1^−/−^ and PD‐1^−/−^xRAG1^−/−^ mice for their production of cytokines. These in vitro‐derived ILC2 were also exposed to agonist and antagonist of PPAR‐γ.

**Results:**

We found that ILC2 from PD‐1^−/−^xRAG1^−/−^ mice had reduced frequencies of IL‐5 and IL‐13 producing cells both in vitro upon IL‐33 stimulation and in vivo following intraperitoneal administration of IL‐33 when compared with ILC2 from RAG1^−/−^ mice. However, by adding IL‐2, IL‐25, and thymic stromal lymphopoietin to the in vitro cultures, the frequency of IL‐5 and IL‐13 expressing ILC2 from PD‐1^−/−^xRAG1^−/−^ mice became similar to the frequency observed for ILC2 from RAG1^−/−^ mice. In addition, PPAR‐γ agonists and antagonists were found to increase and decrease PD‐1 expression on ILC2 respectively.

**Conclusions:**

These findings illustrate that chronic loss of PD‐1 plays a role in ILC2 function and PD‐1 expression can be modulated by PPAR‐γ.

**Abbreviations**
bmILC2: bone marrow–derived ILC2
GITR: glucocorticoid‐induced TNFR‐related protein
ICOS: inducible T‐cell costimulator
ILC: innate lymphoid cell
PPAR‐γ: peroxisome proliferator activated receptor‐γ
ST2: IL‐33R

## 1 INTRODUCTION

Innate lymphoid cells (ILCs) are cytokine‐producing cells that are typically associated with promoting tissue‐specific immunity and as such are found in compartments including the gut, fat, and lungs of mice. ILC becomes functionally specialized during their development to express interferon γ (IFNγ) (ILC1), interleukin 5 (IL‐5)/IL‐13 (ILC2), or IL‐17/IL‐22 (ILC3). These subsets more or less mirror the cytokine‐producing potential of adaptive T helper cells.^1^ While natural killer (NK) cells are known for their cytotoxicity, the other ILCs appear to be involved in secreting cytokines important in host defense. ILC1s like NK cells produce IFN‐γ, however, they only express the T‐bet transcription factor and not Eomes, as NK cells.^2^ ILC3 cells are similar to Th17 cells expressing the ROR‐γt transcription factor.^3^ ILC2 cells like T helper 2 (Th2) cells express GATA3 and are a major source of IL‐5 and IL‐13 within the innate immune system.^4^, ^5^ ILC2 cells express IL‐7Rα (CD127), IL‐33R (ST2), IL‐17RB, and the receptor for thymic stromal lymphopoietin (TSLP). They have been attributed with a number of functions such as host defense against parasitic helminth infections in the gut,^6^, ^7^, ^8^ inducing eosinophilia and airway hyper‐responsiveness^9^, ^10^ as well as regulating fat metabolism^11^ and wound healing.^12^

The programmed cell death protein 1 (PD‐1) is a checkpoint inhibitor of the immune system and is expressed primarily on lymphocytes during an immune response.^13^ On the surface of T cells, PD‐1 functions primarily as an inhibitory molecule by binding its ligands PD‐L1 and PD‐L2. Both of these ligands are induced on immune cells during inflammatory conditions and engagement of PD‐1 on T cells dampens immune responses by reducing activating signals through the T cell receptor and costimulatory receptors.^13^ This has made targeting PD‐1 and its ligands an attractive immunotherapy against cancer, in particular when cancer cells themselves express PD‐L1.^14^ In addition, PD‐L1 and PD‐L2 engagement may regulate Th1 and Th2 immune responses respectively.^15^ Expression of PD‐L2 on subsets of dendritic cells (DC) and macrophages has been associated with inducing Th2 responses.^15^, ^16^, ^17^

Genome‐wide arrays identified a number of molecules that could be used to separate ILC2 from other ILC.^18^ These included many molecules already associated with Th2 responses such as GATA3, IL‐5, IL‐13, and peroxisome proliferator‐activated receptor‐γ (PPAR‐γ). PD‐1 expression by ILC2 was another hallmark separating ILC2 from NK cells and other ILC subsets.^18^ More recently PD‐1 expression was detected early in ILC development, which was thought to play a role in the development of ILC responses and ILC2 could be depleted with anti‐PD‐1 antibody.^19^ In the present study, we have used PD‐1xRAG1^−/−^ mice to examine the effects of PD‐1 deficiency on ILC2. In addition, we also explored a potential mechanism for the control of PD‐1 expression on ILC2 by the nuclear receptor PPAR‐γ.

## 2 MATERIAL AND METHODS

### 2.1 Mice

RAG‐1^−/−^ 20 and PD‐1xRAG1^−/−^ 21 mice on the C57BL/6 background were housed in isolated cages under specific pathogen‐free conditions at the Department of Microbiology, Tumor and Cell Biology and Astrid Fagraeus Laboratories, Karolinska Institutet, Stockholm. All procedures were performed under both institutional and national guidelines (ethical numbers from Stockholm County Council N147/15). Sex and aged match mice were used for all experiments. Mice were chosen randomly for control or treated groups.

### 2.2 Induction of ILC2 in vivo

RAG‐1^−/−^ and PD‐1xRAG1 mice^−/−^ were injected with 300 ng of recombinant mouse IL‐33 (rmIL‐33) (Biolegend, San Diego, CA) intraperitoneal (IP) or intranasal (IN) every day for 3 days, and killed at day 4. Cells from the peritoneal cavity were collected by peritoneal wash after which, the collected cells were examined for surface markers and intracellular cytokines by flow cytometry as mentioned previously. To block PD‐1, anti‐PD‐1 (clone RMP1‐14, Bioxcell, NH) or control rat IgG were injected at 100 ng per mouse 1 day before IL‐33 injection. The blocking of PD‐1 was confirmed by flow cytometry using the same clone of PD‐1. In experiments using papain, mice were injected IN with 10 μg per mouse consecutively for 3 days and mice were killed on day 4.

For cells from the bronchoalveolar lavage (BAL), lungs were washed with two consecutive flushes of the lung with 1 mL phosphate‐buffered saline (PBS). For lymphocyte isolation from the lungs, the lungs were finely chopped and digested with 0.13 U/mL Liberase (Roche, Switzerland) and 10 ug/mL DNase I (Sigma‐Aldrich, UK) for 40 minutes at 37°C. Remaining tissue pieces were mashed through a 100 μM filter and the digestion reaction terminated by the addition of PBS containing 2% FCS (Sigma‐Aldrich) on ice. The cells were run on a lymphoprep gradient for 12 minutes at 2200 RPM. The cells at the interface were collected and used in experiments.

### 2.3 Generation of bone marrow–derived ILC2

ILC2 were generated from BM following a modified protocol used to generate ILC1.^22^ In brief, BM cells were seeded at 1 × 10^6^ cells/mL and cultured in RPMI 1640 medium (HyClone, South Logan, UT) supplemented with 10% fetal bovine serum (Biowhittaker, Verviers, Belgium), 250 IU/mL penicillin and streptomycin solution, 2 mM glutamine, 50 mM 4‐(2‐hydroxyethyl)‐1‐piperazineethanesulfonic acid, 1× nonessential amino acids and 1 mM sodium pyruvate (HyClone), and 50 µM β‐mercaptoethanol (Gibco, Paisley, UK) and supplemented with 100 ng/mL recombinant mouse stem cell factor and 100 ng/mL recombinant mouse FLT3 ligand (ImmunoTools, Friesoythe, Germany). After 4 days, the cells were collected and the CD90^+^ cells were purified using anti‐CD90 beads and magnetic sorting (Miltenyi Biotech). Cells were then stimulated with for further 4 days in fresh medium containing 10 ng/mL rmIL‐7 and rmIL‐33 (ImmunoTools). rmIL‐2, rmIL‐25, and rmTSLP were added also at 10 ng/mL. For 15dΔ12,14‐PGJ_2_‐ (Sigma‐Aldrich) and GW9662‐ (Sigma‐Aldrich) treated ILC2 cultures, the drugs were added to the culture at the same time as IL‐7 and IL‐33 at 1 and 0.9 μM, respectively, and maintained for the duration of the culture.

### 2.4 Flow cytometry

All antibodies were purchased from Becton Dickinson (San Diego, CA), Bioscience (San Diego, CA) or Biolegend (San Diego, CA); anti‐CD90.2 (clone 53‐2.1; BD Biosciences), anti‐CD127 (clone A7R34; BioLegend), anti‐ST2 (clone RMST2‐2; eBioscience), anti‐CD25 (Clone M‐A251; BD Biosciences) anti‐CD11b (clone M1/70; BioLegend), anti‐IL‐5 (clone TRFK5; BioLegend) and anti‐IL‐13 (clone eBio13A; eBioscience), anti‐GITR (DTA‐1; Biolegend), anti‐ICOS (7E.17G9; BD Biosciences), anti‐TIGIT (GIGD7; eBioscience), CD39 (Duha59; Biolegend), anti‐KLRG1 (clone MAFA; Biolegend), anti‐NK1.1 (clone PK136; Biolegend), anti‐GR1 (GR1; Biolegend). For staining with PD‐1, the clones RMP‐14 or 29F.1A2 were used.

For intracellular cytokine staining, cells were stimulated for 2 hours with phorbol 12‐myristate 13‐acetate (PMA) and ionomycin (Sigma‐Aldrich) after which time point monensin and brefeldin A (Biolegend) were added to the cultures and the cells were cultured for further 2 hours. The cells were fixed in 2% formaldehyde for at least 30 minutes before being permeabilized using permeabilization buffer (Biolegend) and incubated with antibodies at room temperature in the dark for 30 minutes. Cells were washed in PBS. For intranuclear staining, FoxP3 fix and permeabilization buffer was used (eBioscience). Flow cytometry was performed on CyAN ADP LX 9‐color flow cytometer (Beckman Coulter, Pasadena, CA) or LSRII (Becton Dickinson). Data were analyzed using FlowJo software (Tree Star Inc, OR).

### 2.5 Statistical analysis

All bar graphs in figures are represented as the mean ± SD. All statistical analysis was performed using the GraphPad Prism software (La Jolla, CA).

## 3 RESULTS

### 3.1 Effect of IN IL‐33 on ILC2 from PD1xRAG1^−/−^ mice

**Figure 1:**
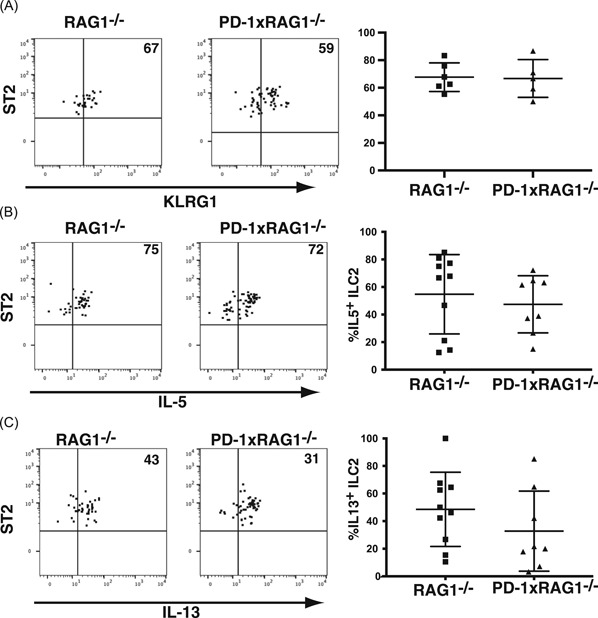
Frequency of KLRG1^+^, IL‐5^+^, and IL‐13^+^ ILC2 from the lungs of RAG1^−/−^ and PD‐1xRAG1^−/−^ mice. A, Frequency of KLRG1^+^ ILC2 from the lungs of untreated RAG1^−/−^ (*n* = 6 mice from a total of two experiments) and PD‐1xRAG1^−/−^ (*n* = 5 mice from a total of two experiments). B, Frequency of IL‐5^+^ ILC2 and (C) IL‐13^+^ ILC2 from the lungs of untreated RAG1^−/−^ and PD‐1xRAG1^−/−^ mice following PMA and ionomycin stimulation. Bar graphs represent mean and SD of data from four separate experiments. PD‐1, programmed cell death protein 1; PMA, phorbol 12‐myristate 13‐acetate; RAG1, recombination‐activating protein 1

**Figure 2:**
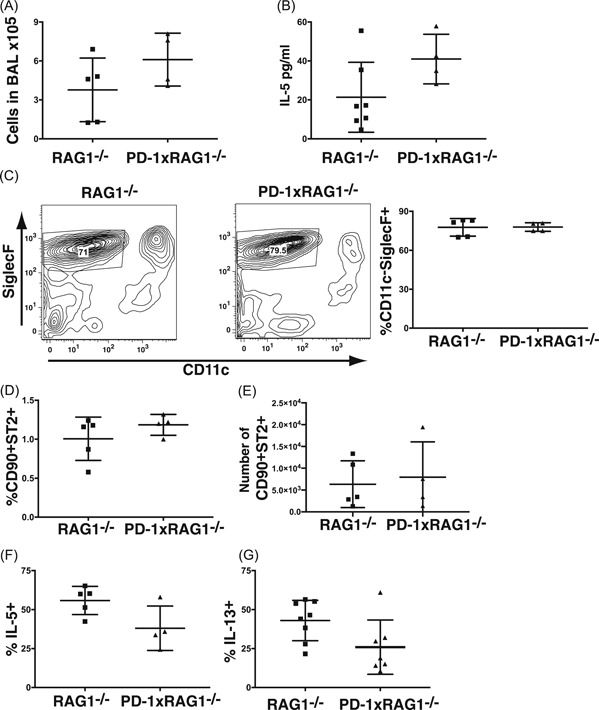
Effect of IN IL‐33 instillation on lung ILC2 from RAG1^−/−^ and PD‐1xRAG1^−/−^ mice. A, Cell numbers from BAL of RAG1^−/−^ and PD‐1xRAG1^−/−^ mice collected after 3 days IN IL‐33 (300 ng per mouse) (*n* = 4‐5 mice, two independent experiments). B, IL‐5 levels in the BAL of RAG1^−/−^ and PD‐1xRAG1^−/−^ mice (*n* = 4‐5 mice, two independent experiments). C, Frequency of eosinophils in the BAL of RAG1^−/−^ and PD‐1xRAG1^−/−^ mice (gated on all live cells), one representative plot is shown, bar graph of all data (*n* = 4‐5 mice, two separate experiments). D, Frequency of ILC2 (CD25^+^CD90^+^ST2^+^) in the BAL of RAG1^−/−^ and PD‐1xRAG1^−/−^ mice (*n* = 4‐5 mice, two separate experiments). E, Numbers of ILC2 in the BAL of BAL of RAG1^−/−^ and PD‐1xRAG1^−/−^ mice (F) Frequency of IL‐5 producing ILC2 (defined as CD11b^−^CD25^+^CD90^+^CD127^+^ST2^+^) from the lungs of RAG1^−/−^ and PD‐1xRAG1^−/−^ (*n* = 4‐5, two separate experiments). G, Frequency IL‐13 producing ILC2 from the lungs of RAG1^−/−^ and PD‐1xRAG1^−/−^ (*n* = 7, three independent experiments). BAL, bronchoalveolar lavage; IL, interleukin; IN,intranasal; PD‐1, programmed cell death protein 1; RAG1, recombination‐activating protein 1; ST2, IL‐33R

ILC2 are a source of type 2 cytokines such as IL‐5 and IL‐13.^5^ PD‐1 is expressed on progenitor ILC2,^19^ and PD‐1 has been identified as a hallmark protein on ILC2.^18^ It has previously been reported the KLRG1^+^ cells are increased in mice lacking PD‐1,^23^ however examining the lungs of RAG1^−/−^ and PD‐1^−/−^xRAG1^−/−^ (PD1xRAG1^−/−^) mice, we found no differences between the frequency of KLRG1^+^ cells (Figure 1A). In addition, we found that the frequency of IL‐5 or IL‐13 expressing ILC2 in the lungs of PD‐1xRAG1^−/−^ mice was equivalent to those found in RAG1^−/−^ mice (Figure 1B,C; gating strategy for ILC2 in Figure S1). When PD‐1xRAG1^−/−^ and RAG1^−/−^ mice were inoculated for three consecutive days with rIL‐33 IN, we found that there were no differences in numbers of cells in the BAL into the airways of PD‐1xRAG1^−/−^ mice when compared with the RAG1^−/−^ mice (Figure 2A). No significant differences in the levels of IL‐5 in the BAL of IL‐33 treated mice were found either (Figure 2B). By gating on live cells in the BAL, there was no difference in the frequency of eosinophils found in the BAL of these mice (Figure 2C), nor was there a striking difference in the frequency or cell numbers of ILC2 between PD‐1xRAG1^−/−^ and RAG1^−/−^ mice (Figure 2D,E). When lung ILC2 were examined, there were no significant differences in the frequency of either IL‐5^+^ ILC2 from PD‐1xRAG1^−/−^ mice (Figure 2F) or IL‐13^+^ ILC2 in the PD‐1xRAG1^−/−^ mice when compared with ILC2 from RAG1^−/−^ mice (Figure 2G). By gating on the IL‐5^+^ and IL‐13^+^ ILC2, we did not find any significant differences in the expression levels of either cytokine between PD‐1xRAG1^−/−^ and RAG1^−/−^ mice (Figure S2).

Papain can induce allergy‐like symptoms in the lungs of mice. When we compared BAL and lungs from mice inoculated IN with papain, we saw a similar pattern of frequency of ILC2 in the lungs of PD‐1xRAG1^−/−^ mice (Figure S3B). Similarly, ILC2 from the lungs of papain treated PD‐1xRAG1^−/−^ mice had similar frequencies of IL‐5^+^ and IL13^+^ cells as ILC2 from RAG1^−/−^ mice (Figure S3C,D).

### 3.2 PD‐1‐deficient ILC2 from peritoneal exudate does not effectively produce cytokines

**Figure 3:**
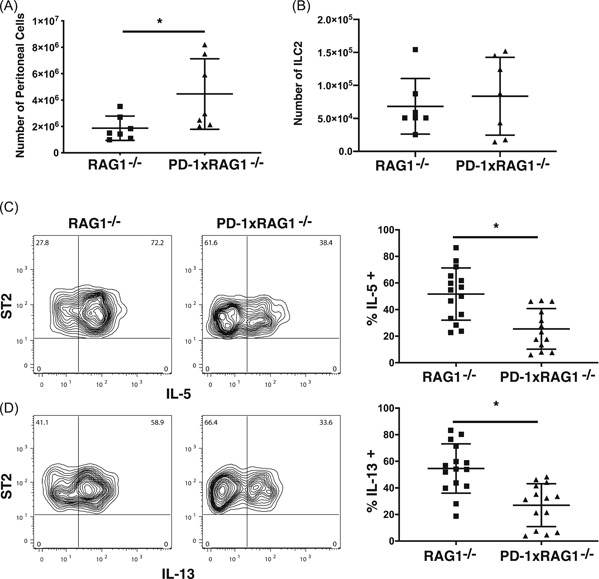
Cytokine production by PD1^−/−^ ILC2 from the peritoneal exudate following IL‐33 IP inoculation. A, Number of cells collected from the peritoneal exudate (*P < .05 Mann‐Whitney test *n* = 7 mice from a total of three independent experiments) and (B) the number of ILC2 from the peritoneum of RAG1^−/−^ and PD‐1xRAG1^−/−^ mice. C, Production of IL‐5 and (D) IL‐13 by ILC2 (defined as CD11b^−^CD25^+^CD90^+^CD127^+^ST2^+)^ from the peritoneal cavity of RAG1^−/−^ and PD‐1xRAG1^−/−^ mice after PMA and ionomycin stimulation. Bar graphs represent mean and standard deviation (*P < .05 Mann‐Whitney test *n* = 14‐15 mice from a total of six independent experiments). ILC, innate lymphoid cell; IP,intraperitoneal; PD‐1, programmed cell death protein 1; PMA, phorbol 12‐myristate 13‐acetate; RAG1, recombination‐activating protein 1; ST2, IL‐33R

To further study the effects of chronic PD‐1 loss on ILC2 in vivo, PD‐1xRAG1^−/−^ mice were injected with rIL‐33 IP for 3 days consecutively. When the peritoneal fluid was collected, there was a significant increase in the number of cells from the peritoneal cavity PD‐1xRAG1^−/−^ mice compared with RAG1^−/−^ mice (Figure 3A), however, this did not lead to a significant increase in the number of ILC2 collected from the mice (Figure 3B). The frequency of IL‐5 expressing ILC2 from the peritoneal cavity of PD‐1xRAG1^−/−^ mice was significantly reduced with approximately 40% less IL‐5^+^ ILC2 than in wildtype mice (27% ± 13% IL‐5^+^ vs 55% ± 20% IL‐5^+^ from ILC2 in RAG1^−/−^ mice, *P* < .05 Mann‐Whitney test; Figure 3C). Similarly, there was an approximately a 30% reduction in the percentage of IL‐13 expressing ILC2 in PD‐1xRAG1^−/−^ mice when compared with ILC2 from RAG1^−/−^ mice (36% ± 10% IL‐13^+^ ILC2 in PD‐1xRAG1^−/−^ mice vs 58% ± 16% from ILC2 in RAG1^−/−^ mice, *P* < .05 Mann‐Whitney test; Figure 3D).

### 3.3 Anti‐PD‐1 antibody treatment of RAG1^−/−^ does not affect cytokine expression in ILC2

**Figure 4:**
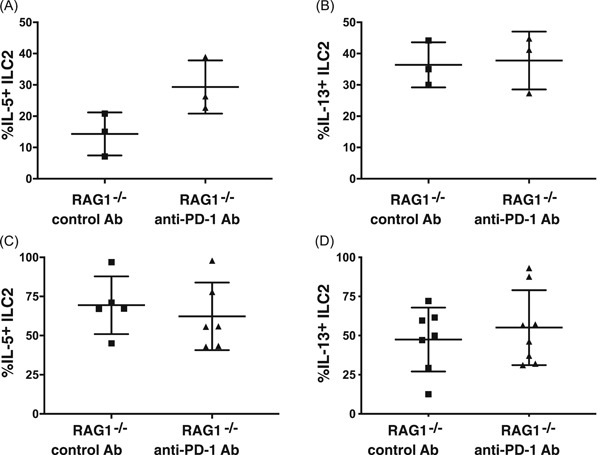
Cytokine expression in ILC2 following anti‐PD‐1 treatment in vivo. Bar graphs represent the frequency of (A) IL‐5 and (B) IL‐13 expressing ILC2 following IN instillation of IL‐33 (*n* = 3). C, Bar graphs represent the percentage of ILC2 expressing IL‐5 and (D) IL‐13 following inoculation IP (*n* = 6‐8, three independent experiments). IL, interleukin; ILC, nnate lymphoid cell; IN, intranasal; IP, intraperitoneal; PD‐1, programmed cell death protein 1

Since PD‐1xRAG1^−/−^ mice have a chronic lack of PD‐1, we also tested whether anti‐PD‐1 antibody treatment of RAG1^−/−^ mice could have an acute effect on ILC2. However, we found no significant difference between the frequencies of IL‐5 expressing ILC2 when mice were treated with anti‐PD‐1 antibody before giving mice IL‐33 IN (14% ± 10% IL‐5^+^ ILC2 in control antibody‐treated RAG1^−/−^ mice vs 29% ± 9% IL‐5^+^ anti‐PD‐1 antibody‐treated RAG1^−/−^ mice; Figure 4A). Similarly, no significant difference in the percentage of ILC2 expressing IL‐13 following anti‐PD‐1 antibody treatment before IL‐33 injection (37% ± 10% IL‐13^+^ ILC2 in control antibody‐treated RAG1^−/−^ mice vs 38% ± 9% IL‐13^+^ anti‐PD‐1 antibody‐treated RAG1^−/−^ mice; Figure 4B). Furthermore, RAG1^−/−^ mice injected IP with IL‐33 and treated with anti‐PD‐1 did not exhibited significant changes in the percentage of IL‐5 (38% ± 21% IL‐5^+^ ILC2 in control antibody‐treated RAG1^−/−^ mice vs 53% ± 24% IL‐5^+^ anti‐PD‐1 antibody‐treated RAG1^−/−^ mice; Figure 4C) or IL‐13 (70% ± 21% IL‐5^+^ ILC2 in control antibody‐treated RAG1^−/−^ mice vs 66% ± 22% IL‐13^+^ anti‐PD‐1 antibody treated RAG1^−/−^ mice; Figure 4D) expressing ILC2.

### 3.4 PD1xRAG1^−/−^ ILC2 from peritoneal exudate does not express exhaustion markers

**Figure 5:**
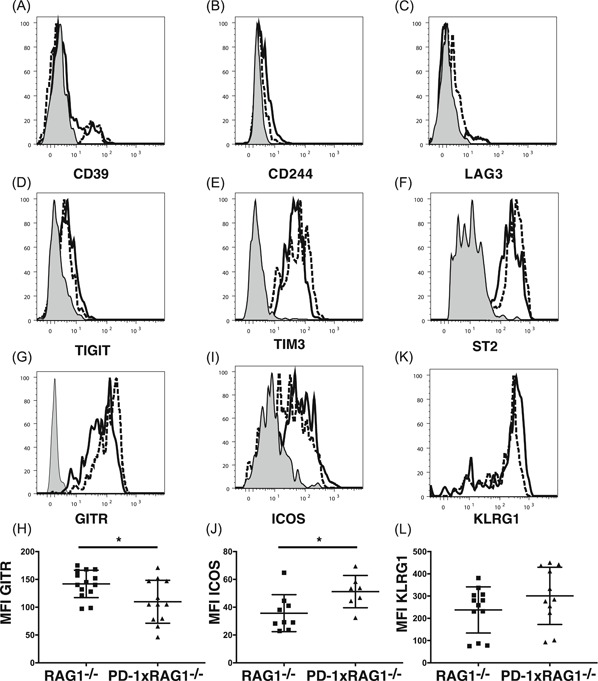
Expression of exhaustion markers, ST2, ICOS, and GITR on ILC2 from the peritoneum following IL‐33 inoculation. Expression of (A) CD39, (B) CD244, (C) LAG3, (D) TIGIT, (E) TIM3, and (F) ST2 on the surface of ILC2 (defined as CD11b^−^CD25^+^CD90^+^CD127^+^ST2^+^) from RAG1^−/−^ (dotted line) and PD‐1xRAG1^−/−^ (solid line) in the peritoneum (control staining shaded line). G, Expression of GITR on the surface of ILC2 from RAG1^−/−^ (dotted line) and PD‐1xRAG1^−/−^ (solid line) mice (control staining shaded line). H, Comparison of GITR expression between RAG1^−/−^ (squares) and PD‐1xRAG1^−/−^ (triangles) mice. Bar graphs represent results from 11 to 12 mice from four independent experiments (*P < .05 Mann Whitney test). I, Expression of ICOS on the surface of ILC2 from RAG1^−/−^ (dotted line) and PD‐1xRAG1^−/−^ (solid line) mice in the peritoneum (control staining shaded line). J, Comparison of ICOS expression between RAG1^−/−^ (squares) and PD‐1xRAG1^−/−^ (triangles) mice. Bar graphs represent results from 7 to 9 mice from three independent experiments (*P < .05 Mann Whitney test). K, Expression of KLRG1 on the surface of ILC2 from RAG1^−/−^ (dotted line) and PD‐1xRAG1^−/−^ (solid line) mice in the peritoneum (control staining shaded line). L, Comparison of KLRG1 expression between RAG1^−/−^ (squares) and PD‐1xRAG1^−/−^ (triangles) mice (*n* = 11‐12 mice). Bar graphs represent results from four separate experiments. GITR, glucocorticoid‐induced TNFR‐related protein; ICOS, inducible T‐cell costimulator; IL, interleukin; PD‐1, programmed cell death protein 1; RAG1, recombination‐activating protein 1; ST2, IL‐33R

One possibility for the decrease in cytokine production by the ILC2 from PD1xRAG1^−/−^ mice is that chronic lack of PD‐1 expression by these cells led to their exhaustion. Therefore, we examined the expression levels of CD39, CD244, LAG3, TIGIT, or TIM3, which have all been associated with PD‐1 and T cell exhaustion.^24^ However, we did not see any significant changes in the expression of these molecules between the ILC2 from RAG1^−/−^ and PD‐1xRAG1^−/−^ mice (Figure 5A‐E). The expression levels of ST2 might have an effect on cytokine production but there was no difference in ST2 expression between ILC2 from RAG1^−/−^ and PD‐1xRAG1^−/−^ mice (212 mean fluorescence intensity [MFI] ± 84 vs 218 MFI ± 99; Figure 5F).

### 3.5 Decreased expression of GITR on ILC2 from PD‐1xRAG1^−/−^ mice in vivo

ILC subsets also express several members of the TNF receptor superfamily.^18^, ^25^ The glucocorticoid‐induced TNFR‐related protein (GITR) is such a member and has been associated with immune‐suppression particularly on T_reg_. GITR‐GITRL interactions were found to enhance Th2 responses in helminthic infection and potentiated Th2 responses in a model of airway hyper‐responsiveness.^26^, ^27^ Following IL‐33 treatment, there was a significant reduction in expression of GITR on the ILC2 from PD‐1xRAG1^−/−^ mice when compared with ILC2 from RAG1^−/−^ mice (92 MFI ± 34 vs 131 MFI ± 21, respectively, *P* < .05 Mann Whitney test, Figure 5G,H).

### 3.6 Increased T‐cell costimulator protein expression on PD‐1 deficient ILC2 in vivo

The inducible T‐cell costimulator protein (ICOS) has been identified as an important molecule in ILC2 homeostasis. The lack of ICOS ligand leads to both reduced KLRG1 expression on ILC2 and cytokine production by ILC2.^28^ Since we had seen reduced cytokine production in the ILC2 from PD‐1xRAG1^−/−^ mice, we investigated if this was due to reduced ICOS expression. However, ILC2 from PD‐1xRAG1^−/−^ mice expressed higher levels of ICOS than ILC2 from RAG1^−/−^ mice (51 MFI ± 11 vs 36 MFI ± 13, respectively, *P* < .05 Figure 5I,J). When we examined KLRG1 expression on the ILC2 from PD‐1xRAG1^−/−^ mice, there was slightly higher expression but it was not significantly different from the expression on ILC2 from RAG1^−/−^ mice (271 MFI ± 156 vs 202 MFI ± 123, respectively, Figure 5K,L). Thus, ICOS expression did not play a role in the reduced expression of Th2 cytokines in the ILC2 from the PD‐1xRAG1^−/−^ mice.

### 3.7 Generation of ILC2 from bone marrow

**Figure 6:**
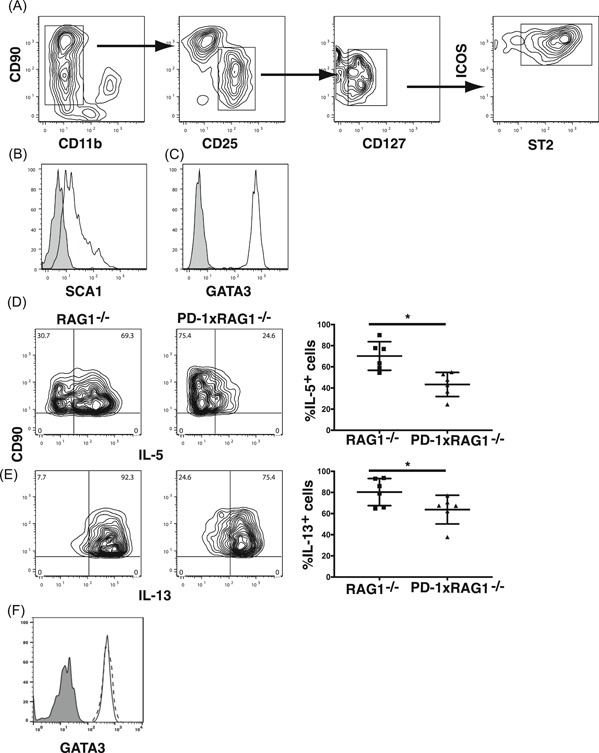
Generation of bmILC2. A, Phenotype of ILC2 generated from bone marrow cells following incubation of CD90 enriched cells with IL‐7 and IL‐33 for 4 days. Expression of (B) SCA1 and (C) GATA3 on bmILC2 (control antibody shaded line) (D) bmILC2 from PD1xRAG1^−/−^ mice express less IL‐5 and (E) IL‐13 when compared bmILC2 generated from RAG1^−/−^ mice following stimulation with PMA and ionomycin. Fluorescence‐activated cell sorting plots represent one experiment, bar graph represents data from all experiments (*P < .05 Mann Whitney test from six independent cultures). F, Expression of GATA3 is not altered in bmILC2 from PD1xRAG1^−/−^ mice, (solid line) or bmILC2 from RAG1^−/−^ mice (dashed line). bmILC2, bone marrow–derived ILC2; IL, interleukin; ILC, innate lymphoid cell; PD‐1, programmed cell death protein 1

To examine ILC2 function in vitro, we developed a culture system to generate ILC2 from mouse bone marrow cells. Bone marrow cells were first incubated in Flt3 ligand and stem cell factor for 4 days after which they were selected for CD90 expression and recultured in rIL‐7 and rIL‐33 for a further 4 days. At the end of the 4 days, we found both CD90^high^CD11b^−^ cells and CD90^int^CD11b^−^ cells in the cultures. However, only the CD90^int^CD11b^−^ cells expressed markers associated with ILC2 including CD127, SCA1, CD25, and ST2 (IL‐33R) (Figure 6A,B). Furthermore, these cells expressed GATA3, further demonstrating that these cells were ILC2 (Figure 6C). Similar to our observations in vivo, bone marrow‐derived‐ILC2 (bmILC2) generated from PD1xRAG1^−/−^ mice had a noticeable decrease in the frequency of IL‐5 and IL‐13 expressing cells when compared with wild type bmILC2 (Figure 6D,E). The reduced frequency of cytokine‐producing ILC2 from PD1xRAG1^−/−^ mouse was not due to alterations in GATA3 expression, because the expression of GATA3 was equivalent in bmILC2 generated from both RAG1^−/−^ and PD1xRAG1^−/−^ (Figure 6F).

**Figure 7:**
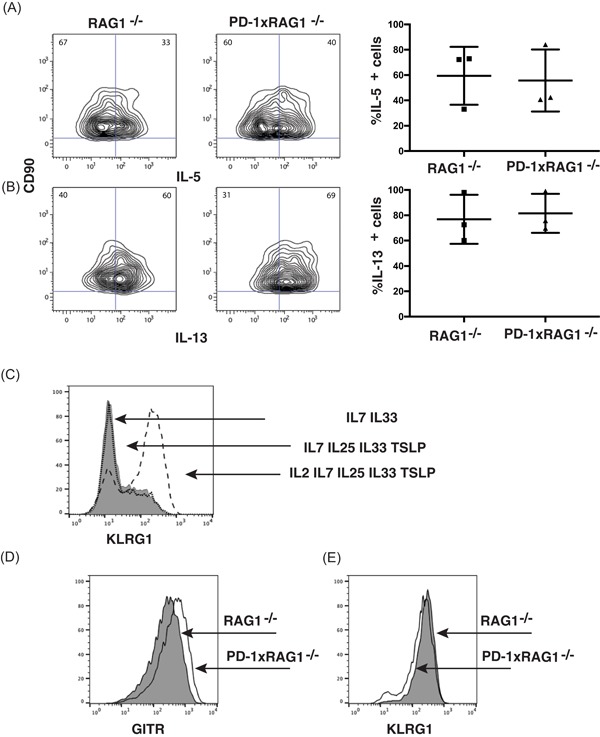
Stimulation of bmILC2 with IL‐2, IL‐7, IL‐25, IL‐33, and TSLP recovers cytokine production in PD‐1 deficient ILC2. A, IL‐5^+^ and (B) IL‐13^+^ bmILC2 from RAG1^−/−^ mice and PD1xRAG1^−/−^ mice following stimulation with IL‐2, IL‐25, and TSLP in addition to IL‐7 and IL‐33. Data is from three independent cultures. C, IL‐2 increases KLRG1expression on bmILC2. Cell were cultured in IL‐7 and IL‐33 (shaded histogram plot), IL‐7, IL‐25, IL‐33, and TSLP (solid line) or IL‐2, IL‐7, IL‐25, IL‐33, and TSLP (dashed line) for 4 days after CD90 enrichment. D, Expression of GITR in bmILC2 from RAG1^−/−^ mouse (shaded histogram plot) and PD1xRAG1^−/−^ mouse (solid line). E, Expression of KLRG1 in bmILC2 from RAG1^−/−^ mouse (shaded histogram plot) and PD1xRAG1^−/−^ mouse (solid line). bmILC2, bone marrow–derived ILC2; GITR, glucocorticoid‐induced tumor necrosis factor receptor; IL, interleukin; PD‐1, programmed cell death protein 1; TSLP, thymic stromal lymphopoietin

While IL‐33 is important for the development of bmILC2, other cytokines such as IL‐25 and TSLP have been shown to also be important for ILC2 in vivo.^29^, ^30^, ^31^ Furthermore, IL‐2 is important in stimulating IL‐5 and IL‐13 production by ILC2.^32^ When bmILC2 were stimulated with IL‐2, IL7, IL‐25, IL‐33, and TSLP, there was now no difference in the frequency of cytokine‐producing bmILC2 from RAG1^−/−^ and PD1xRAG1^−/−^ (Figure 7A,B). This suggested that these cytokines recover the production of IL‐5 and IL‐13 in the PD‐1 deficient ILC2. Furthermore, by adding IL‐2 to the cultures, the frequency of KLRG1^+^ bmILC2 increased in the cultures when compared with bmILC2 stimulated with IL7, IL‐25, IL‐33 and TSLP or IL‐7 and IL‐33 (Figure 7C). However, when comparing bmILC2 from RAG1^−/−^ and PD1xRAG1^−/−^ mice generated in the presence of IL‐2, IL7, IL‐25, IL‐33, and TSLP, we did not observe major changes in the expression of GITR (Figure 7D) nor KLRG1 (Figure 7E). This suggested that in vivo GITR downregulation in PD1xRAG1^−/−^ mice maybe due to other factors, for example, interaction with its ligand.

### 3.8 PPAR‐γ promotes PD‐1 expression on ILC2

**Figure 8:**
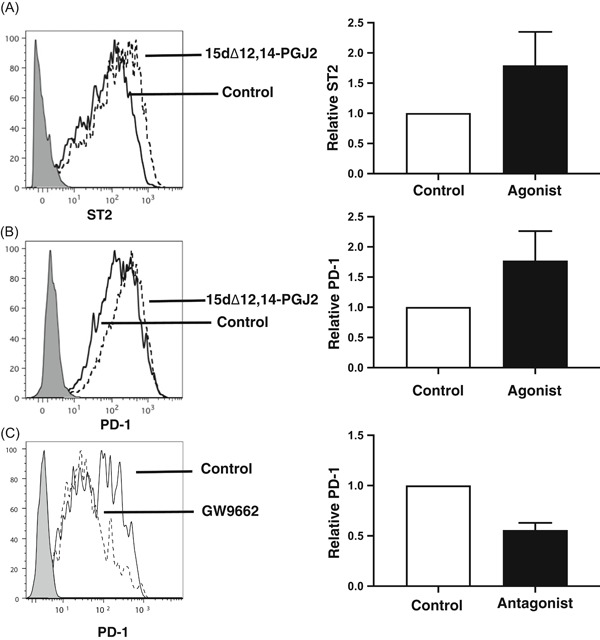
PPAR‐γ controls expression of PD‐1 on ILC2. A, Expression of ST2 on bmILC2 is increased after exposure to PPAR‐γ agonist 15dΔ12,14‐PGJ_2_. Bar graph represents relative expression of ST2 from seven independent experiments. B, Expression of PD‐1 on bmILC2 is increased after exposure to PPAR‐γ agonist 15dΔ12,14‐PGJ_2_. Bar graph represents relative expression of PD‐1 from seven independent experiments. C, Expression of PD‐1 on ILC2 is decreased after exposure to PPAR‐γ antagonist GW9662. Bar graph represents relative expression of PD‐1 from four independent experiments. bmILC2, bone marrow–derived ILC2; ILC, innate lymphoid cell; PD‐1, programmed cell death protein 1; PPAR‐γ, peroxisome proliferator‐activated receptor‐γ; ST2, IL‐33R

PPAR‐γ is a master regulator of adipocyte differentiation and a potent modulator of lipid metabolism,^33^ a suppressor of proinflammatory cytokine secretion,^34^ and has been being associated with Th2 cell immune responses.^35^, ^36^, ^37^ In addition, PPAR‐γ has also been identified as a hallmark for ILC2.^18^ Similar to our previous findings,^35^ when the PPAR‐γ agonist 15dΔ12,14‐PGJ_2_ (PGJ2) was added to the bmILC2 cultures, there was almost a twofold increase in the expression levels of ST2 on the surface of ILC2 cultured with PGJ2 compared with bmILC2 cultured in the rIL‐7 and rIL‐33 alone (MFI ST2 115 ± 79 for control cultured vs 214 ± 158 for PGJ2 cultured ILC2, *P* < .05 paired *t* test; Figure 8A). When the expression levels of PD‐1 were examined, we found that expression of PD‐1 was almost doubled on the bmILC2 that were stimulated with PGJ2 (MFI PD‐1 245 ± 121 vs 449 ± 280, *P* < .005 paired *t* test, *n* = 7; Figures 8B and S4A). Similar observations were also made using another PPAR‐γ agonist, pioglitazone (Figure S4B). To further demonstrate that PPAR‐γ might affect PD‐1 expression, the PPAR‐γ antagonist, GW9662, was added to the cultures. Expression of PD‐1 was reduced by almost 45% on the ILC2 cultured with GW9662 compared with the control ILC2 (PD‐1 MFI 148 ± 51 vs 83 ± 31, *P* < .005 paired *t* test, *n* = 5; Figures 8C and S4C). Expression of GITR was not altered between cells treated with PGJ2 or GW9662 when compared with control‐treated cells (Figure S4D). These data suggested that PPAR‐γ could be a regulator of PD‐1 expression on ILC2.

## 4 DISCUSSION

In the present study, we found that chronic loss of PD‐1 expression on ILC2 could affect the induction or maintenance of the production of IL‐5 and IL‐13 in vivo. In addition, other costimulatory molecules GITR and ICOS also had altered expression in the chronic absence of PD‐1. We could also demonstrate that PD‐1 expression on ILC2 could be controlled by PPAR‐γ. These data suggested that the expression of PD‐1 on ILC2 might have physiological significance for not only ILC2 development but also for ILC2 function.

Our results are slightly at odds with a previous publication that demonstrated that KLRG1^+^PD‐1^+^ ILC2 produce more Th2 cytokines following both IN delivery of IL‐33 and during infection with the worm *Nippostrongylus brasiliensis*.^23^ In our study, we found that there were no differences between ILC2 from RAG1^−/−^ and PD‐1xRAG1^−/−^ mice in their expression of KLRG1. Furthermore, we did not see increases in cytokine production in the ILC2 from PD‐1xRAG1^−/−^ mice either in vitro or in vivo, but the levels were either reduced or similar when compared with RAG1^−/−^ mice. The reduction in the frequency of cytokine producing ILC2 from PD‐1xRAG1^−/−^ mice was most pronounced when IL‐33 was delivered into the peritoneum. One possible reason for these discrepancies between our study and that of Taylor et al^23^ is that we used mice lacking RAG genes and these mice lack both T and B cells that might have feedback effects on ILC. In the study of Taylor et al,^23^ IL‐33 could still interact with T cells expressing ST2,^38^ which might skew immune responses.^39^ In addition, it has been found that IL‐2 can increase IL‐5 and IL‐13 production by ILC2^32^ and IL‐2 would be an abundant cytokine in immunocompetent mice. In RAG1^−/−^ mice only ILC3s were found to be a major source of IL‐2.^32^ Adding IL‐2 to our cultured ILC2 could mature the ILC2 because the number of cells expressing KLRG1 increased and the cultures of RAG1^−/−^ and PD‐1xRAG1^−/−^ ILC2 showed no differences in the frequency of IL‐5 or IL‐13 expressing ILC2. Therefore, IL‐2 from T cells in immunocompetent mice might also play a role in maturation of ILC2 which could explain the differences between our findings and those of Taylor et al.

Although Taylor et al^23^ treated *N brasiliensis* infected RAG1^−/−^ mice with anti‐PD‐1 antibody and saw increased cytokine production and protection, we did not see any discernable differences in the frequency of cytokine‐producing cells when mice were treated with IL‐33 IN or IP in combination with anti‐PD‐1 antibody. In our studies, we concluded that chronic loss of PD‐1 might have a greater effect on the ILC2 rather than an acute blocking of PD‐1. However, we cannot rule out that microbiome differences between animal facilities could also explain differences between our study and Taylor et al.^40^ For example, the mice in our study are maintained in IVC cages from birth and so these mice may have limited exposure to microbes which might alter immune responses.

From our study, the reasons for why chronic PD‐1 deficiency might reduce Th2 cytokine production could be explained by two hypotheses, which are not mutually exclusive. The first hypothesis centers on the recent finding that PD‐1 is important for long‐term responsiveness and function of memory CD8 T cells. In this study, Odorizzi et al^41^ demonstrated that CD8^+^ T cells from mice lacking PD‐1 produced less IFNγ and TNF 42‐ and 300‐days postinfection with lymphocytic choriomeningitis virus than their WT counterparts. These authors hypothesized that PD‐1 was important in preserving exhausted T cells from overstimulation, excessive proliferation, and terminal differentiation. Thus, in our study, it could be rationalized that PD‐1 deficient ILC2 became too exhausted to make cytokines following prolonged exposure to IL‐33. While possible, this would be a rapid realization of this effect of PD‐1 on immune function, that is, within 3 days. Since anti‐PD‐1 antibody treatment did not significantly affect cytokine production by ILC2 in RAG1^−/−^ mice, this suggested that chronic lack of PD‐1 might affect ILC2 development/activation rather than acute blocking with anti‐PD‐1. However chronic lack of PD‐1 did not lead to any increase in the expression of markers associated with T cell exhaustion such as CD39, LAG3, TIM3, or TIGIT on the PD‐1 deficient ILC2, which were observed on CD8^+^ T cells lacking PD‐1.^41^

An alternative hypothesis is that PD‐1/PD‐L2 interactions might be important in maintaining type 2 cytokine production by ILC2. PD‐L2 expression on DCs is important in driving Th2 responses^15^, ^17^ and more recently it was shown that ILC2 feedback on DC is important for driving Th2 memory responses.^42^ Therefore, one can imagine that the lack of PD‐1/PD‐L2 interaction between DC and ILC2 could inhibit the feedback loops necessary to create a strong ILC2 response. Future studies using mice deficient in PD‐L1 or PD‐L2 could shed light on whether the ligands for PD‐1 can affect ILC2 functions. Indeed, PD‐L1 expression on ILC2 has recently been shown to be important in maintaining/inducing Th2 responses through PD‐1 on Th2 cells.^43^

In a house dust mite antigen model for allergy, mice lacking PD‐1 were found to have exacerbated allergy responses compared with wild‐type mice.^44^ In this model, the authors found that the PD‐1 deficient CD4^+^ T cells produced diminished levels of Th2 cytokines. This reduction in Th2 cytokines is therefore similar to our findings in the ILC2 cells. Since ILC2 are the early source of Th2 cytokines and could potentially affect CD4 T cells^43^ just as NK cells have been found to affect adaptive immune responses,^45^ it is tempting to speculate that reduced Th2 cytokine production by PD‐1 deficient ILC2 might skew the CD4 T cell responses in PD‐1 deficient mice.

Cells in adipose tissue express high levels of PD‐1 and PPAR‐γ,^46^, ^47^ suggesting that the interplay, whether direct or indirect, between these two molecules might play important role in fat metabolism. PD‐1 can play a role in controlling T cell metabolism,^48^ thus it is tempting to hypothesize that the PPAR‐γ‐PD‐1 axis might also help to control cellular metabolism. The fact that we observed that PPAR‐γ could modulate PD‐1 and ST2 expression on ILC2 might imply two scenarios. In the first scenario, PPAR‐γ has direct effects on both PD‐1 and ST2 independently of each other, while the second scenario would imply that PPAR‐γ by affecting the expression of either ST2 or PD‐1 then affects the expression of the other. In support of the first scenario, we have found in the present study that lack of PD‐1 does not affect ST2 expression on ILC2 and furthermore increased expression of ST2 induced by the short‐chain fatty acid butyrate or histone deacetylase inhibitors does not correspondingly lead to increased PD‐1 expression on the ILC2 (Figure S5). Thus if PPAR‐γ can directly affect PD‐1 expression, antagonists of PPAR‐γ might be useful in directing antitumor immune responses where PD‐1 interactions with its ligands play a role in dampening immune responses to tumors.^14^ Therefore, the interplay between PPAR‐γ and PD‐1 in inflammation relating to cancer and metabolic syndromes is an area that should be further explored.

In the present study, we have demonstrated that chronic loss of PD‐1 expression on ILC2 might have a role in maintaining cytokine production in these cells and expression of receptors associated with ILC2 function. Since ILC2 can express PD‐1^23^, ^25^ and with current anti‐PD‐1 or anti‐PD‐L1 therapies for the treatment of cancers,^14^ it would be interesting to examine whether some of the functions associated with ILC2 are also affected in these patients. For example, skewing of Th2 cytokines might have both beneficial as well as detrimental effects on tumor therapies.^49^

## Supporting information

Supporting information

## Data Availability

All the data presented here are new and fully accessible.
