# Supplementary material for: PD‐1 expression affects cytokine production by ILC2 and is influenced by peroxisome proliferator‐activated receptor‐γ
Source: Immun Inflamm Dis. 2019 Nov 19;8(1):8–23. doi: 10.1002/iid3.279 (PMC7016838; doi:10.1002/iid3.279)
Supplement: Supplementary file 1 — Supporting information [file IID3-8-8-s001.pdf]

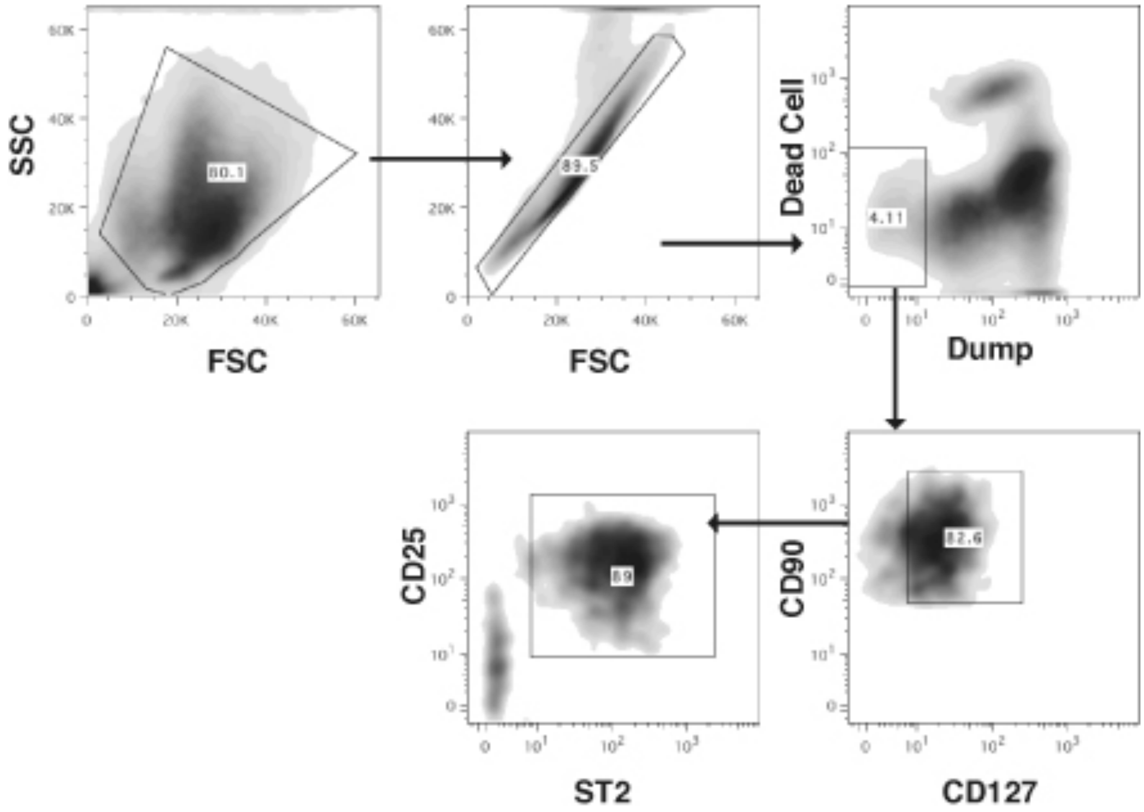

Supplemental Figure 1. Gating for ILC2 in vivo. Cells were first sorted on living cells that were negative for CD11b, GR1 and NK1.1. After this, cells were gated on CD90 and CD127 expression and finally the cells were further gated on CD25 and ST2 expression. These cells were then used to determine expression of cytokines and other surface markers. Plots in figure come from staining of cells collected from the peritoneum but similar gating was used for ILC2 from the lungs.

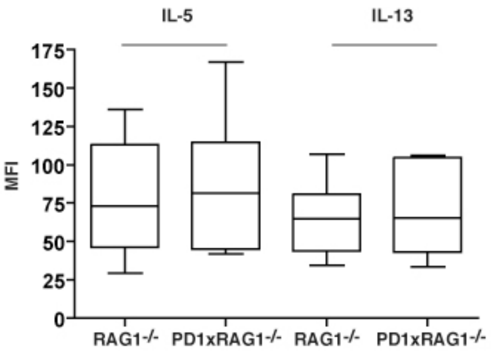

Supplemental Figure 2. Expression levels of IL-5 and IL-13 from ILC2. Gating on the IL-5^{+} or IL-13^{+} ILC2, the expression levels were compared between RAG1$^{-/-}$ and PD1xRAG1$^{-/-}$

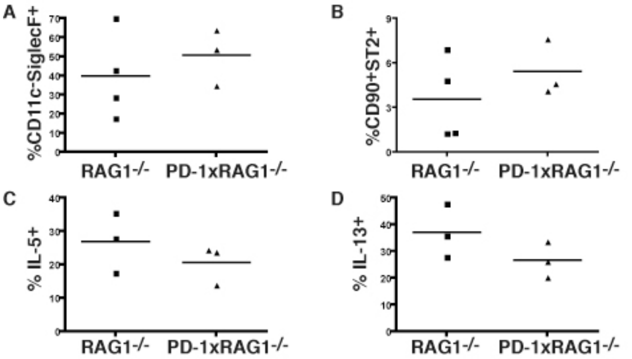

Supplemental Figure 3. Intranasal inoculation with papain induces inflammation in the lung of RAG1$^{-/-}$ and PD1xRAG1$^{-/-}$ mice. (A) Frequency of eosinophils in the BAL of RAG1$^{-/-}$ and PD-1xRAG1$^{-/-}$ mice collected after 3 days i.n papain (10µg/ mouse) (B) Frequency of ILC2 in the BAL of RAG1$^{-/-}$ and PD-1xRAG1$^{-/-}$ mice. (C) Frequency of IL-5 producing ILC2 (defined as CD11b^{-}CD25^{+}CD90^{+}CD127^{+}ST2^{+}) from the lungs of RAG1$^{-/-}$ and PD-1xRAG1$^{-/-}$. (D) Frequency IL-13 producing ILC2 from the lungs of RAG1$^{-/-}$ and PD-1xRAG1$^{-/-}$.

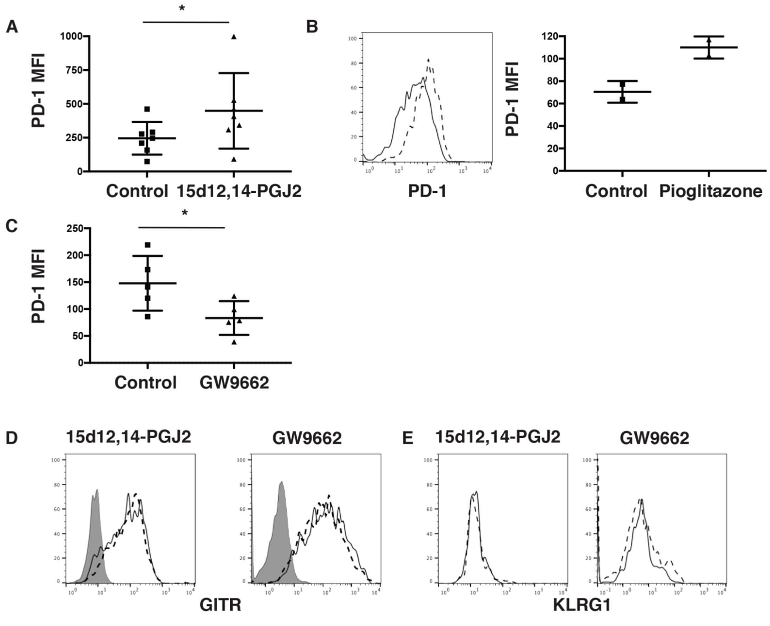

Supplemental Figure 4. Expression levels of PD-1 following incubation with PPARγ agonists and antagonists. (A) MFI of PD-1 expression following incubation with PGJ2, ($p<0.05$ paired t-test, n=7). (B) *Left*, histogram plot of PD-1 expression on ILC2 following incubation with pioglitazone (*dashed line* pioglitazone treated and *solid line* control treated bmILC2). *Right,* bar graph of MFI of PD-1 from two separate experiments comparing pioglitazone and control treated bmILC2. (C) MFI of PD-1 expression on GW9662 treated and control treated bmILC2 ($p<0.005$ paired t-test, n=5). (D) Histogram plots of GITR expression from bmILC2 treated with (*left*) PGJ2 or (*right*) GW9662. (*dashed line* PGJ2 or GW9662 treated and *solid line* control treated ILC2) (E) Histogram plots of KLRG1 expression from bmILC2 treated with (*left*) PGJ2 or (*right*) GW9662. (*dashed line* PGJ2 or GW9662 treated and *solid line* control treated ILC2)

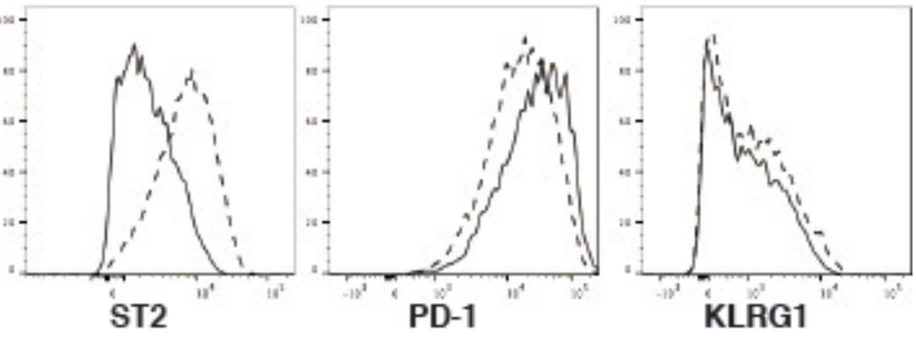

Supplemental Figure 5. Expression of ST2, PD-1 and KLRG1 on bmILC2 after stimulation with the short chain fatty acid, butyrate. *Solid lines*, control treated bmILC2. *Dashed lines* butyrate-treated bmILC2
